# Deaths Caused by Malignant Pustule and Lyssa in Italy in 1893 and 1894

**Published:** 1896-12

**Authors:** 


Deaths Caused by Malignant Pustule and Lyssa in Italy in 1893 and 1894. According to a report of the "Rivista d'igiene e sanità publica" the number of deaths from malignant pustule in Italy in 1894 reached 645, and in 1893, 598. From lyssa 93 persons died in 1894, and 83 in 1893.—*Thierärztliches Centralblatt*, February 15, 1896.

